# Chronic Stress Modulates Microglial Activation Dynamics, Shaping Priming Responses to Subsequent Stress

**DOI:** 10.3390/brainsci15050534

**Published:** 2025-05-21

**Authors:** Junyu Chen, Jiacheng Huang, Taolei Han, Nobuhiko Kojima

**Affiliations:** 1 Laboratory of Molecular Neurobiology, Faculty of Life Sciences, Toyo University, Saitama 351-8510, Japan; chen.junyu.r2@dc.tohoku.ac.jp (J.C.); huangjiacheng190@gmail.com (J.H.);; 2 Super-Network Brain Physiology, Graduate School of Life Sciences, Tohoku University, Sendai 980-8577, Japan; 3 Department of Histology and Cell Biology, Graduate School of Medical Sciences, Kanazawa University, Kanazawa 920-8640, Japan; 4 Research Center for Biomedical Engineering, Toyo University, Saitama 351-8510, Japan

**Keywords:** microglia, microglial priming, stress vulnerability, depression, chronic unpredictable mild stress (CUMS)

## Abstract

(1) Background: The high recurrence rate and individual differences in stress susceptibility contribute to the diverse symptoms of depression, making full recovery and relapse prevention challenging. Emerging evidence suggests that fluctuations in microglial activity are closely linked to depression progression under chronic stress exposure. Changes in the brain microenvironment can elicit microglial priming, enhancing their sensitivity to external stimuli. However, few studies have longitudinally examined how microglial characteristics evolve throughout depression progression. (2) Methods: In this study, we investigated microglial morphological changes and their responses to acute stress at different stages of depression using the chronic unpredictable mild stress (CUMS) paradigm in mice. (3) Results: Our findings reveal that in the dentate gyrus, microglial activation indices, including cell number and morphology, exhibit distinct dynamic patterns depending on CUMS exposure duration. Notably, after 2 and 4 weeks of CUMS exposure followed by acute stress re-exposure, microglia display opposing response patterns. In contrast, after 6 weeks of CUMS exposure, primed microglia exhibit dysfunction, failing to respond to acute stress. Notably, depressive behaviors are not prominent after 2 weeks of CUMS exposure but become more pronounced after 4 and 6 weeks of exposure. Additionally, regardless of CUMS duration, body weight demonstrates an intrinsic capacity to normalize after stress cessation. (4) Conclusions: These findings suggest that microglial priming responses are state-dependent, either enhancing or suppressing secondary stimulus responses, or exceeding physiological limits, thereby preventing further activation. This study provides novel insights into the role of microglial priming in stress vulnerability and its contribution to depression progression.

## 1. Introduction

Depression is a highly recurrent psychiatric disorder characterized by low mood and anhedonia, yet its comprehensive pathological mechanisms, particularly those related to relapse vulnerability, remain unclear [1]. Emerging evidence has been revealed that microglia play a crucial role in the progression of depression. As a key subset of innate immune cells in the central nervous system (CNS), microglia interact with neurons, astrocytes, and other cells to maintain brain homeostasis. Their functions include synaptic pruning, neurite formation, microenvironmental monitoring, programmed cell death, and phagocytosis of cellular debris [2,3]. Microglia are dynamic cells that respond rapidly to stress, undergoing changes in number, morphology, and function, particularly in stress-sensitive brain regions such as the medial prefrontal cortex and hippocampus in rodent models [4,5,6]. However, the temporal dynamics of microglial activity during the transition from acute (short-term) to chronic (long-term) stress remain poorly understood.

Additionally, depression relapse rates increase with the frequency of prior episodes, suggesting that prolonged stress may lead to chronic inflammation, thereby aggravating vulnerability and ultimately contributing to mood disorders [7,8]. Recent studies have highlighted the phenomenon of microglial priming, triggered by physiological factors such as aging, inflammatory stimuli, and stress, which can alter microglial function. This state of primed activation increases susceptibility to secondary stimuli, potentially triggering an intensified pro-inflammatory response in a shorter timeframe [9,10]. However, the relationship between microglial priming and stress-induced vulnerability, particularly under chronic stress exposure, remains unclear.

Therefore, this study employed a well-established rodent model of depression, the chronic unpredictable mild stress (CUMS) paradigm. Exposure to various unpredictable stressors disrupts homeostasis, causes weight loss, and induces depression-like states. Accumulating evidence suggests that this model closely resembles the behavioral and physiological variables observed in patients with major depressive disorders [11,12]. This study aimed to elucidate the temporal dynamics of microglial changes following short-term and long-term CUMS exposure and further investigate microglial priming by examining morphological changes during re-exposure to acute stress after different durations following the initial stress. Our findings suggest that the fluctuation pattern of microglial priming is determined by the current state of microglial activation, which varies depending on the intensity and duration of chronic stress exposure, ultimately influencing subsequent response to acute stress. These findings highlight that microglial responses to chronic stress are not always enhanced but rather depend on the intensity and duration of stress, and in some cases, microglial activity can become diminished.

## 2. Materials and Methods

### 2.1. Animals

A total of 36 male C57BL/6N mice (wild type, 8 weeks old, 21 ± 2 g) were purchased from Oriental Yeast Co., Ltd. (Tokyo, Japan). The animals were housed in groups of 6 per cage and kept in a Multi-chamber Animal Housing System (Nippon Medical & Chemical Instruments Co., Ltd., Osaka, Japan) under standard conditions, including a 12 h light/dark cycle (lights on between 6 a.m. and 6 p.m.), a temperature of 23 ± 1 °C, and ad libitum access to food and water. All animal experiments in this study adhered to the National Institutes of Health Laboratory Animal Care and Use Guidelines (NIH Publication No.80-23) and were approved by the Animal Experiment Committee of Toyo University Itakura Campus (Gunma, Japan). Approval details for the experiments are as follows: approval code: University-wide Protocol 2022-30 (approval date: 1 April 2022); approval code: University-wide Protocol 2023-26 (approval date: 1 April 2023).

### 2.2. Stress Exposure Procedures

#### 2.2.1. Stable Chronic Unpredictable Mild Stress Exposure Paradigm

**Figure 1:**
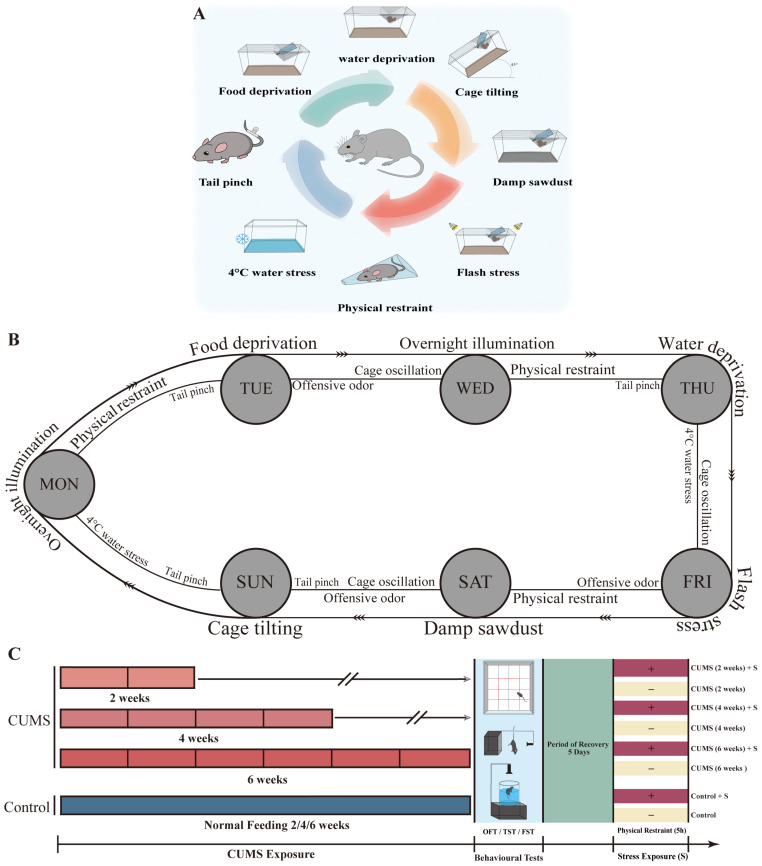
CUMS paradigm and weekly exposure schedule. (**A**) An illustration of the 11 stressors used in the CUMS paradigm, Table 2. The CUMS frequencies per week are indicated. (**B**) These stressors were randomly administered to the CUMS group mice each week based on the exposure schedule. The outer part of the exposure schedule represents prolonged stressors, such as flash exposure (24 h), whereas the inner section represents short-term stressors, such as tail pinching (2 min). (**C**) A schematic timeline of the experimental procedures.

Some modifications were made to the chronic unpredictable mild stress (CUMS) protocol administered to mice, as previously described [13,14]. The following 11 stressors were used in the CUMS groups: tail pinch, 4 °C water stress, cage oscillation, offensive odor, 3 h of physical restraint, 12 h of overnight illumination, 15 h of water deprivation, 15 h of food deprivation, 24 h of flash, 24 h of damp sawdust, and 24 h of cage tilting (Table 1). Mice were randomly exposed to three to four stressors per day in a 7-day cycle and received the same amount of stress each week (Table 2). The mice were divided into CUMS groups (with exposure durations of 2, 4, and 6 weeks; three groups) and their respective control groups (control for 2, 4, and 6 weeks; three groups) (details shown in Figure 1A–C).

#### 2.2.2. Acute Stress (Re-)Exposure Before Sacrifice

After the CUMS model was completed, behavioral tests were conducted, and the mice were allowed to recover for 5 days. On the day of tissue collection, the mice in each group were randomly divided into two subgroups (three mice per subgroup), with one subgroup being subjected to physical restraint stress for 5 h (Figure 1C). For the CUMS group, this served as stress re-exposure, whereas acute stress exposure was considered for the control group. Finally, subgroups that experienced physical (re-)restraint were given 1.5 h to recover before sacrifice.

### 2.3. Behavioral Testing

#### 2.3.1. Open Field Test (OFT)

An open field test (OFT) was conducted to evaluate locomotor activity, exploratory behavior, and anxiety levels. The bottom of the open field apparatus (40 cm length × 40 cm width × 30 cm height) was divided into 25 equal squares, with nine squares in the middle defined as the center region (36% of the overall area). Each mouse was placed in the same corner of the apparatus and allowed to freely explore it for a 10 min duration. The apparatus was illuminated at a uniform intensity of 50 lux. The activity of each mouse was recorded using software (TimeOFCR1 ver.20130327, O’Hara & Co., Ltd., Tokyo, Japan). The anxiety index and locomotion behavior were evaluated based on the total moving distance and the ratio of time spent in the center region. Each mouse’s track plot was obtained and analyzed offline using AxoGraph (AxoGraph Scientific, Sydney, Australia). Before testing the next mouse, the apparatus was sterilized with 75% alcohol.

#### 2.3.2. Tail Suspension Test (TST)

The tail suspension test (TST) has been widely used to assess depression- and despair-like behaviors in mice. The front end of each mouse’s tail was secured with adhesive tape and then suspended, maintaining the nose at 20 cm above the desktop. A camera was placed perpendicular to the mouse at a distance of 32 cm to record the duration of immobility over a 6 min period. The data were analyzed using software (TimeFZ1 ver.20120926, O’Hara & Co., Ltd., Tokyo, Japan). Immobility was defined as the mice remaining completely still without struggling and the absence of any limb or body movements

#### 2.3.3. Forced Swimming Test (FST)

The forced swimming test (FST) has been used to identify depressive/despair-like behaviors in mice. Each mouse was placed in a clear cylindrical container (12 cm diameter, 22 cm height) filled with 15.5 cm of water (22 ± 1 °C) and tested for 6 min. A camera placed above the cylinder was used to record the duration of immobility for each mouse, which was then analyzed using software (TimeFZ1, O’Hara & Co., Ltd., Tokyo, Japan). Immobility was defined as the absence of any active movement by the mouse, except for what was necessary to keep the head above the water.

### 2.4. Immunohistochemical Assay

The animals were anesthetized with an injection of sodium pentobarbital (150 mg/kg, i.p.) and sacrificed after all tests. Intracardial perfusion with phosphate-buffered saline (PBS) containing 0.5% heparin was performed to remove blood, and the brains of the mice were fixed with 4% paraformaldehyde (PFA). The fixed brains were sectioned into coronal slices (40 µm thick) using a vibratome (LEICA VT 1200S, Nussloch, Germany). Slices containing the medial prefrontal cortex (mPFC) and hippocampal regions were collected and stored in 24-well plates with 0.05% NaN_3_ in 1 × PBS at 4 °C.

#### 2.4.1. Diaminobenzidine Staining with Iba-1 Antibodies

Selected sections containing the mPFC and hippocampus (dentate gyrus, DG) were used for 3,3′- diaminobenzidine (DAB) staining to detect microglial density in each region. Sections were mounted onto slides and treated with 0.3% H_2_O_2_ in 1 × PBS for 30 min at room temperature (RT). Subsequently, they were blocked in blocking buffer [(5% Blocking Ace, 0.1% Triton X-100, and 0.05% NaN_3_) in 1 × PBS] for 1h at RT. The sections were then incubated with the primary antibody, rabbit anti-ionized calcium-binding adapter molecule 1 (Iba1, Wako Chemicals, Richmond, VA, USA, Cat.No.019-19741, 1:800), overnight at 4 °C, followed by incubation with a biotinylated goat anti-rabbit antibody (Vector Labs, Cat.No.BA-1000, 1:200) for 1 h at RT. The sections were then treated with Vectastain Elite ABC reagent (Vector Labs, Newark, CA, USA, Cat.No.PK-6100) for 30 min at RT and stained with a DAB Substrate Kit (Vector Labs, Cat.No.SK-4100). Images were captured using a fluorescence microscope (BX63; Olympus, Tokyo, Japan). For the mPFC, three sections were selected for each mouse and at least two images were captured from each section (a minimum of 18 images per group). For the DG, each mouse had three sections, with at least one image captured from each section (a minimum of nine images per group). Each image in the mPFC was subdivided into two parts (300 µm × 300 µm, individually), and those in the DG region into three parts (250 µm × 250 µm, individually) to calculate cell density. All images were randomized for blinding, and manual cell counting was conducted using the Fiji/ImageJ software (2.16.0, National Institutes of Health, Bethesda, MD, USA).

#### 2.4.2. Immunofluorescence Staining with Iba-1 Antibodies

As previously described, microglia are highly dynamic cells that continuously remodel their morphology in response to environmental changes [5]. In this study, immunofluorescence staining was used to analyze microglial morphological alterations that were characterized by changes in the number of branches in response to varying durations of chronic unpredictable mild stress (CUMS) exposure. Sections containing the hippocampus (dentate gyrus, DG) were blocked with M.O.M Mouse IgG Blocking Reagent (Vector Labs, Cat.No.BMK-2202) containing 0.1% Triton X-100 and 0.05% NaN_3_ in 1 × PBS for 1 h at RT. They were then incubated overnight at 4 °C with the primary antibody, rabbit anti-Iba1 (Wako Chemicals, Cat.No.019-19741, 1:800). The secondary antibody, donkey anti-rabbit IgG (H + L) Alexa Fluor^®^ 594 (Invitrogen, Carlsbad, CA, USA, Cat.No.A21207, 1:200), was applied for 1 h at RT. Subsequently, 4′6-diamidino-2-phenylindole (DAPI, 1:1000) was added and incubated for 15 min at RT. Images were obtained using a laser scanning confocal super-resolution microscope (SpinSR10; OLYMPUS, Tokyo, Japan). Three sections were selected from each mouse and at least two images were captured per section (a minimum of 18 images per group). Although few tools were available in the past two decades for accurately quantifying microglial morphological changes that occur in response to fluctuations in neuronal activity or in the local ionic microenvironment, several reliable image-based analytical methods have been developed over the past ten years [15,16]. These approaches have significantly improved the accuracy and reproducibility of assessing microglial morphological remodeling in fixed tissue. Following the protocols described in these studies, the microglial branch number was quantified using Fiji/ImageJ. The images were skeletonized and analyzed using the AnalyzeSkeleton (2D/3D) plugin. Quantitative data were subsequently processed using a customized Python (3.8) script based on Pandas (v1.0.5), NumPy (v1.18.5), and SciPy (v1.5.0).

### 2.5. Statistical Analysis

All data presented in this study are presented as mean ± standard error of the mean (SEM). All statistical analyses, including *p*-values, were performed using GraphPad Prism software (version 10.0, San Diego, CA, USA) and are reported as four significant figures excluding *p*-values in this study. The two groups were compared using a two-sided unpaired *t*-test with Welch’s correction if the assumption of equal variance was violated. One-way ANOVA followed by Tukey’s post-hoc test was used to compare more than two groups if the variance among the groups was significant. Two-way ANOVA followed by Bonferroni’s post-hoc test was used to compare the effects of the two factors, and all statistical measure details are presented in Table S1. Simple linear regression was performed to analyze the correlation between the two change indices, with the R^2^ value and *p*-value reported. Statistical significance was set at *p* < 0.05. Specific details regarding the statistical tests, sample sizes, and *p*-values are described in the figure legends.

## 3. Results

### 3.1. Body Weight Gain Was Suppressed During CUMS Exposure

**Figure 2:**
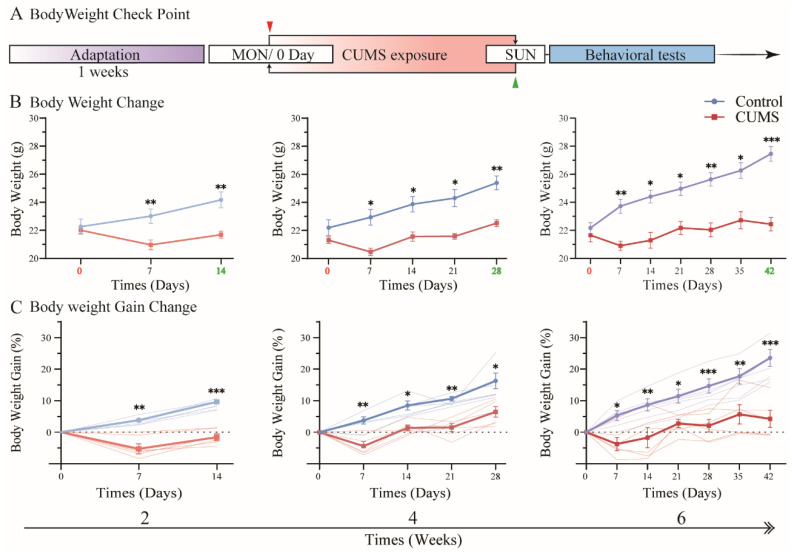
CUMS exposure induced a remarkable reduction in the body weight of mice. (**A**) The body weights of all mice were measured on the first day (day 0, red arrow) of commencing the CUMS exposure procedure. Subsequently, body weight was measured every Sunday (green arrow) each week. (**B**) All CUMS group mice showed a significant decrease in body weight compared to their respective control group mice (Table S1A). (**C**) The body weight gain of all CUMS group mice was suppressed and significantly lower than that of the respective control group (Table S1B). Statistical analysis was performed using two-way ANOVA, followed by Bonferroni’s post-hoc test. Data are presented as the mean ± SEM, n = 6 for each group. * *p* < 0.05, ** *p* < 0.01, *** *p* < 0.001.

To determine how changes in mouse body weight are affected by chronic unpredictable mild stress (CUMS) during different exposure durations, the mice were assigned to three groups and individually exposed to CUMS for 2, 4, or 6 weeks (Figure 2A). In this context, mice in the control groups were also fed normally without CUMS exposure for 2, 4, or 6 weeks, respectively. To minimize baseline weight variability, all mice were allowed to feed normally for one week prior to CUMS exposure for adaptation, and the initial body weight (baseline, day 0) was recorded upon CUMS exposure. Notably, there was no significant difference in initial body weight among the groups. Several studies have indicated that depressive symptoms are associated with various physiological and behavioral changes. Interestingly, these symptoms are generally stable, except for variations in body weight gain. The effects of chronic physical or social stress on body weight, typically leading to weight loss, are commonly observed in CUMS or SDS (social defeat stress) mice [17,18]. However, some studies have reported no significant weight loss in CUMS mice, despite clear manifestations of other depression-like behaviors [19,20].

In our study, CUMS exposure significantly affected body weight in all CUMS groups, with differences emerging as early as the first week compared to their respective controls. Although body weight in all CUMS groups showed a relatively sharp reduction after the first week of CUMS exposure compared with subsequent weeks, it was generally maintained around baseline levels (day 0) throughout CUMS exposure. After CUMS exposure, all CUMS group mice exhibited a significant reduction in body weight compared to their respective controls (Figure 2B; 14 days: control = 24.17 ± 1.30 g vs. CUMS = 21.68 ± 0.56 g, *p* = 0.006; 28 days: control = 25.38 ± 1.11 g vs. CUMS = 22.52 ± 0.55 g, *p* < 0.001; 42 days: control = 27.45 ± 1.16 g vs. CUMS = 22.43 ± 1.89 g, *p* < 0.001; mean ± SD detailed in Table S1A). Moreover, a significant reduction in body weight was observed in all CUMS groups compared to their respective control groups, suggesting that chronic stress exposure, such as CUMS, can suppress body weight gain in mice (Figure 2C, detailed in Table S1B).

Figure 2 suggests that CUMS-induced suppression of body weight gain is strongly influenced by several factors, including exposure duration, stress intensity, and the type of stressors applied. Furthermore, these findings suggest that the CUMS protocol used in our study consistently and stably induced body weight suppression while minimizing systemic dysfunction. Interestingly, during chronic stress exposure, an acute response was observed in the initial stage, which was followed by an adaptive resistance to chronic stress, even in terms of body weight gain.

### 3.2. Body Weight Gain Increased Tremendously After CUMS Exposure

**Figure 3:**
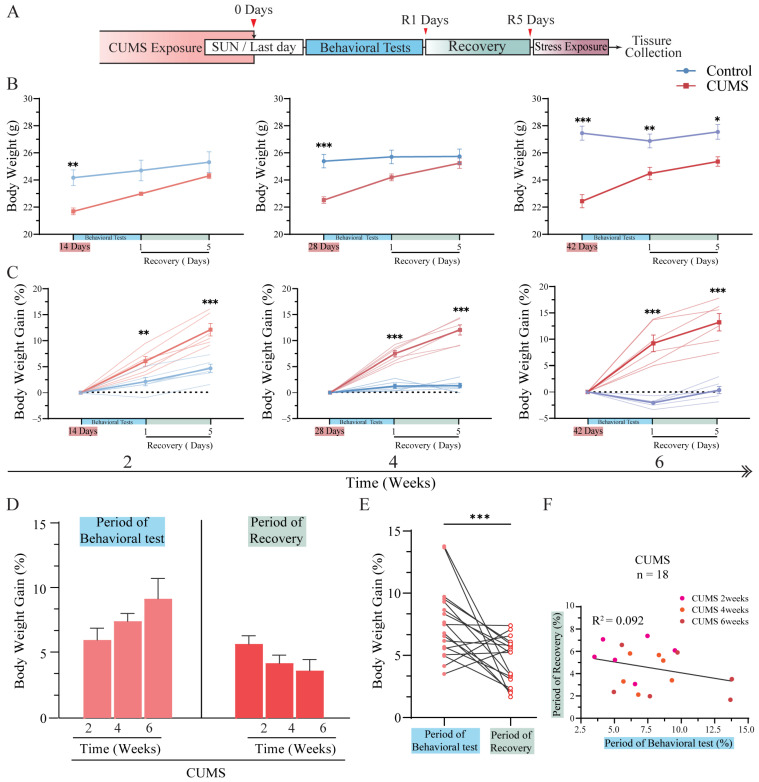
The body weight of the mice rapidly recovered after the cessation of CUMS exposure. (**A**) The body weight of all mice was measured on Sunday of the final week and regarded as day 0 upon exposure to CUMS (day 0, red arrow). After the behavioral test, the body weight of all mice was measured on the final day of the test and on the final day of the recovery period, which were regarded as recovery day 1 (R1, red arrow) and recovery day 5 (R5, red arrow), respectively. (**B**) The body weight of the CUMS group mice sharply increased to match the control group level after 2 and 4 weeks of CUMS exposure, whereas a significant difference was observed after 6 weeks of CUMS exposure compared to the respective control group, as assessed by two-way ANOVA followed by Bonferroni’s post-hoc test (Table S1C, n = 6 for each group). (**C**) The body weight gain of all CUMS group mice was significantly higher than that of the control group mice, as assessed by two-way ANOVA followed by Bonferroni’s post-hoc test (Table S1D, n = 6 for each group). (**D**) No significant difference in body weight gain among all CUMS group mice during the behavioral test and recovery periods was observed, as assessed by one-way ANOVA followed by Tukey’s post-hoc test (F(2,15) = 2.057, *p* = 0.16 during the behavioral test period; F(2,15) = 2.231, *p* = 0.14 during the recovery period, n = 6 for each group). (**E**) The body weight gain during the behavioral test was significantly higher than during the recovery period in all CUMS groups, as assessed by Student’s two-tailed paired *t*-test (t(34) = 3.747, *p* < 0.001, total CUMS group mice (n = 18)). (**F**) No correlation was found between body weight gain during the behavioral test and the recovery period in all CUMS group mice, as assessed by simple linear regression (R^2^ = 0.092, *p* = 0.222, total CUMS group (n = 18)). Data are presented as the mean ± SEM, * *p* < 0.05, ** *p* < 0.01, *** *p* < 0.001.

As mentioned earlier (Figure 2), body weight gain in all CUMS groups was already suppressed and significantly lower than in the control groups. This fluctuation pattern in body weight gain is consistent with previous studies [17,21,22]. However, the post-exposure fluctuation pattern of body weight gain following chronic stress (e.g., CUMS) remains unclear. To investigate this, all CUMS and control group mice were allowed to feed normally for approximately five days to recover after CUMS exposure and behavioral tests. In this analysis, the body weight on the last day of CUMS exposure was considered the baseline (day 0), while measurements were also taken on the first day (recovery day 1, R1) and the last day (recovery day 5, R5) of the recovery period following behavioral tests (Figure 3A).

After the cessation of CUMS exposure and a five-day recovery period, body weight in CUMS group mice increased rapidly despite undergoing behavioral tests such as the forced swim test (FST) and tail suspension test (TST), both of which are considered acute stressors in mice. Notably, except for the 6-week CUMS group, body weight at R5 reached levels comparable to their respective control groups, with no significant differences observed (Figure 3B, left and middle; R5 of 2 weeks: control = 25.32 ± 1.72 g vs. CUMS = 24.30 ± 0.45 g, *p* = 0.56; R5 of 4 weeks: control = 25.73 ± 1.23 g vs. CUMS = 25.23 ± 0.86 g, *p* > 0.99; mean ± SD). In contrast, while body weight in the 6-week CUMS group also increased significantly, it remained notably lower than that of the 6-week control group (Figure 3B, right; R5 of 6 weeks: control = 27.55 ± 1.22 g vs. CUMS = 25.37 ± 0.80 g, *p* = 0.01; mean ± SD detailed in Table S1C).

The body weight gain of all CUMS groups was noticeably higher than that of their respective control groups after CUMS exposure and exhibited a similar pattern of fluctuation across all CUMS groups (Figure 3C, detailed in Table S1D). Moreover, no significant differences in body weight gain were observed among the CUMS groups during behavioral testing and recovery (Figure 3D). The results in Figure 3D suggest that body weight fluctuations induced by CUMS exposure, lasting up to six weeks, remained within the limits of systemic physiological function. Additionally, the ability to recover from CUMS-induced weight loss was preserved. Notably, the rate of body weight recovery appeared relatively stable, maintaining a consistent level regardless of the duration of CUMS exposure, as long as it remained within the limits of systemic physiological function.

However, body weight gain during the behavioral test phase was greater than that observed during the recovery period (Figure 3E). This finding indirectly suggests that, although behavioral tests can induce acute stress, they may not be as intense as the stress caused by CUMS exposure. Consequently, the observed body weight gain following CUMS exposure can be considered a direct reflection of the recovery process, gradually stabilizing before reaching a level comparable to that of the control group. Interestingly, as shown in Figure 3D, despite maintaining a similar level of regained body weight after CUMS exposure (with the last day of CUMS exposure as the baseline), body weight gain appeared to show a negative correlation between the behavioral test period and the recovery period. However, no significant correlation was ultimately observed between these two periods (Figure 3F). These results demonstrate that chronic stress exposure affects body weight during the exposure period. Nevertheless, upon cessation of stress exposure, mice exhibited a rapid increase in body weight, even after six weeks of CUMS exposure, ultimately reaching levels comparable to those of their respective control groups.

### 3.3. Long-Term CUMS Exposure Induced Depression-like Behavior in Mice, but Not in the Short Term

**Figure 4:**
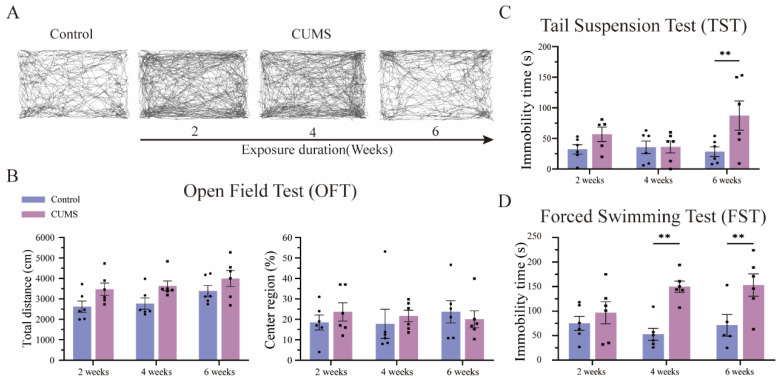
CUMS exposure induced depression-like behavior, which depended on the duration of exposure. (**A**) No differences were observed when comparing traces of all CUMS group mice with control group mice. (**B**) No significant difference was found between all CUMS group mice and their respective control group mice in either the total distances traveled or the ratio of time spent in the center region (Table S1E,F). (**C**) The immobility time in the TST was significantly increased in the 6-week CUMS group mice compared to their respective control group mice (Table S1G). (**D**) The immobility time in the FST was significantly increased in the 4- and 6-week CUMS groups compared to their respective control groups (Table S1H). Statistical analysis was performed using two-way ANOVA, followed by Bonferroni’s post-hoc test. Data are presented as the mean ± SEM, n = 6 for each group. ** *p* < 0.01.

To evaluate the effects of different durations of CUMS exposure on mice, anxiety-like behavior was assessed using the open field test (OFT), while depression-like behavior was measured using the tail suspension test (TST) and forced swim test (FST). The OFT is widely used to evaluate locomotor activity and anxiety-like behavior [23]. Therefore, we compared the total distance traveled and the ratio of time spent in the center region between the different CUMS groups and their respective control groups in the OFT (Figure 4A). Contrary to expectations, there was no significant decrease in the ratio of time spent in the center region between CUMS-exposed mice and their respective controls (Figure 4B, right; detailed in Table S1E). Moreover, no significant difference in total distance traveled, an indicator of locomotor activity, was observed between CUMS-exposed and control mice (Figure 4B, left; detailed in Table S1F). These findings indicate that CUMS exposure does not appear to induce anxiety-like behavior in mice, regardless of exposure duration.

The FST and TST are widely used to assess depression-like behaviors in animals [24,25]. Therefore, we performed FST and TST to compare immobility time across different CUMS exposure durations. No significant difference in immobility time was observed between CUMS-exposed and control mice after 2 and 4 weeks of CUMS exposure. However, after 6 weeks of CUMS exposure, a significant increase in TST immobility time was detected (Figure 4C; 6-week immobility time: control = 28.33 ± 17.60 s vs. CUMS = 87.33 ± 53.43 s, *p* = 0.010; mean ± SD, left; detailed in Table S1G). Similarly, in the FST, no significant difference in immobility time was observed between CUMS-exposed and control mice after 2 weeks of exposure. However, immobility time significantly increased after 4 and 6 weeks of CUMS exposure (Figure 4D; 4-week immobility time: control = 52.67 ± 27.24 s vs. CUMS = 149.83 ± 25.79 s, *p* = 0.002; 6-week immobility time: control = 71.20 ± 44.21 s vs. CUMS = 153.17 ± 50.89 s, *p* = 0.004; mean ± SD, detailed in Table S1H).

A comparison of TST and FST results suggests that short-term CUMS exposure (2 weeks), which can be considered a relatively “acute” chronic stress paradigm, does not induce detectable depression-like behavior. In contrast, long-term CUMS exposure (4 and 6 weeks) appears to induce typical depression-like behavior in mice. These findings suggest that prolonged CUMS exposure is required to elicit significant depression-like symptoms in mice.

### 3.4. The Density of Microglia Showed Dynamic Variations in the Hippocampus After Different Durations of CUMS Exposure

**Figure 5:**
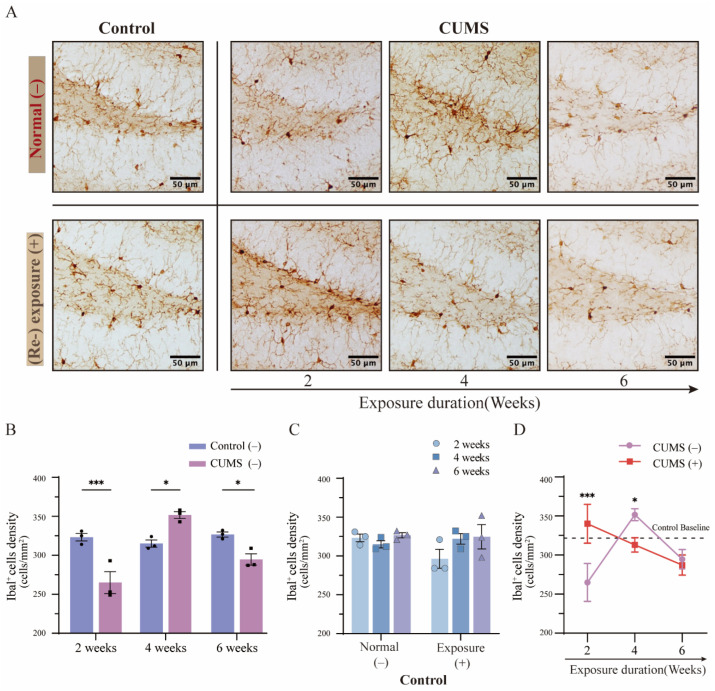
Different durations of CUMS exposure and subsequent acute stress re-exposure led to dynamic variations in microglial density in the hippocampus. (**A**) DAB staining of Iba-1 images showed changes in microglial density in the DG of the hippocampus in the control group mice and mice after different durations of CUMS exposure (top panel). Additionally, changes in microglial density after CUMS exposure followed by acute stress (re-)exposure are shown in the bottom panel. Images show only the left side of the DG, n = 3 for each group; scale bar = 50 µm. (**B**) Microglial density showed different fluctuation patterns after different durations of CUMS exposure (Table S1I). (**C**) No significant difference in microglial density was observed between the control group mice exposed to acute stress and those not exposed (Table S1J). (**D**) After CUMS exposure followed by acute stress re-exposure, the microglial density exhibited a converse fluctuation pattern compared to the respective CUMS group mice not exposed to acute stress (Table S1K). Statistical analysis was performed using two-way ANOVA, followed by Bonferroni’s post-hoc test. Data are presented as the mean ± SEM. Each sample was obtained from at least 18 images (n = 3 for each group); * *p* < 0.05, *** *p* < 0.001.

The hippocampus has been widely recognized as a key structure not only for memory formation and spatial navigation but also for regulating emotional responses. Additionally, hippocampal lesions and impaired neurogenesis are strongly associated with the development of depression-like symptoms [26]. Recent studies suggest that hippocampal pathology is closely linked to neuroinflammation, with microglia playing a central role in the immune response within the central nervous system [27]. In this study, to investigate microglial activation in the hippocampus following different durations of CUMS exposure, microglial density was evaluated using immunohistochemical DAB staining for Iba-1 (Figure 5A, top panel; an overview image is shown in Figure S1). Notably, all CUMS-exposed mice underwent a 5-day recovery period before tissue collection, during which they were not subjected to (re-)acute stress exposure via physical restraint.

Under normal brain homeostasis, microglial density remains relatively stable, which is consistent with our observations in the control hippocampus across the 2-week-to-6-week experimental period (Figure 5B, blue column; detailed in Table S1I). In contrast, compared to their respective control groups, CUMS-exposed mice exhibited a significant reduction in hippocampal microglial density after 2 weeks of exposure, followed by a marked increase after 4 weeks and a subsequent decline after 6 weeks (Figure 5B, red column; 2 weeks: control = 323.33 ± 17.84 vs. CUMS = 265.0 ± 19.87 cells/mm^2^, *p* < 0.001; 4 weeks: control = 315.0 ± 6.49 vs. CUMS = 351.67 ± 6.34 cells/mm^2^, *p* = 0.01; 6 weeks: control = 326.67 ± 4.50 vs. CUMS = 294.67 ± 18.14 cells/mm^2^, *p* = 0.03; mean ± SD). These findings suggest that hippocampal microglial density undergoes dynamic changes depending on the duration of CUMS exposure.

### 3.5. The Phenomenon of Microglial Priming Showed a Converse Pattern Depending on the Duration of CUMS Exposure

Stress vulnerability has long been linked to the onset and relapse of depression, with both susceptibility genes and environmental factors contributing to its development [28,29]. However, the relationship between fluctuations in microglial activation and modifications in stress vulnerability remains unclear. To address this, half of the mice in each group (including the control group) were subjected to 5 h of physical restraint as an acute stress (re-)exposure before tissue collection. Microglial density in the hippocampus was then assessed and compared using immunohistochemical DAB staining for Iba-1 (Figure 5A, bottom panel; an overview image is shown in Figure S1). Previous studies have reported that acute stress exposure activates microglia and induces morphological changes as early as 1 h post-exposure, while the overall number of microglia remains unchanged [30]. Consistent with this, hippocampal microglial density in control mice remained stable despite acute stress exposure (physical restraint), showing no significant changes (Figure 5C; detailed in Table S1J).

Interestingly, following 2 weeks of CUMS exposure and subsequent acute stress re-exposure, a significant increase in hippocampal microglial density was observed compared to the non-re-exposed 2-week CUMS group. Moreover, this increase was statistically significant compared to the respective control groups (Figure 5D; 2 weeks: CUMS (−) = 265.0 ± 19.87 vs. CUMS (+) = 340.0 ± 20.41 cells/mm^2^, *p* < 0.001; mean ± SD). Conversely, after 4 weeks of CUMS exposure followed by the same acute stress re-exposure, microglial density decreased significantly compared to the non-re-exposed 4-week CUMS group, with no significant difference from the respective control group (Figure 5D; 4 weeks: CUMS (−) = 351.67 ± 6.34 vs. CUMS (+) = 313.0 ± 7.48 cells/mm^2^, *p* = 0.05; mean ± SD). However, after 6 weeks of CUMS exposure and additional acute stress re-exposure, no significant difference was observed compared to the non-re-exposed 6-week CUMS group, while a substantial reduction remained in comparison to the respective control group (Figure 5D; 6 weeks: CUMS (−) = 294.67 ± 18.14 vs. CUMS (+) = 287.33 ± 18.54 cells/mm^2^, *p* > 0.99; mean ± SD; detailed in Table S1K). These findings suggest that the priming response of microglial activation in the hippocampus follows a dynamic and fluctuating pattern depending on the duration of chronic stress exposure.

### 3.6. The Density of Microglia Showed Relatively Stable Variations in the mPFC After Different Durations of CUMS Exposure

**Figure 6:**
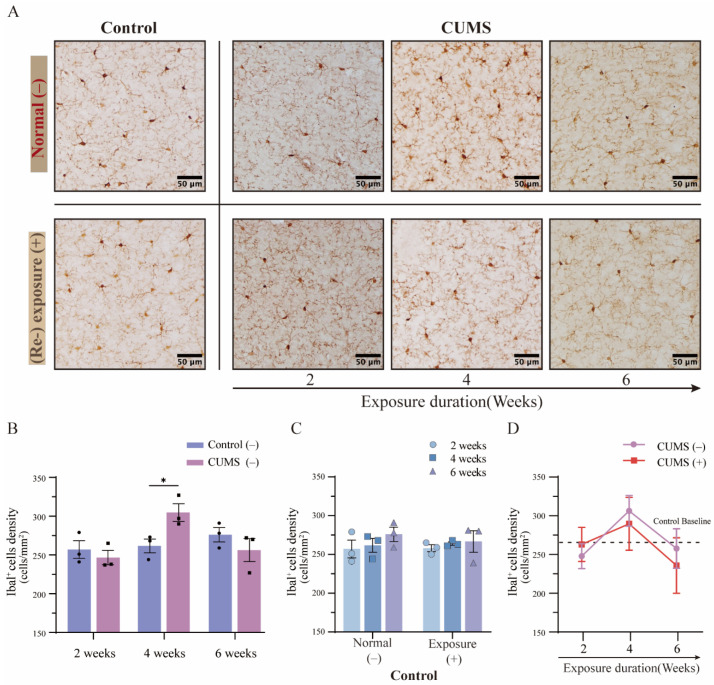
The microglial density showed a significant increase after 4 weeks of CUMS exposure in the mPFC, and no significant difference was observed after CUMS exposure followed by acute stress re-exposure. (**A**) DAB staining of Iba-1 images showed changes in microglial density in the mPFC of the control group mice and mice after different durations of CUMS exposure (top panel). Additionally, changes in microglial density after CUMS exposure followed by acute stress (re) exposure are shown in the bottom panel. Scale bar = 100 µm. (**B**) A significant increase in microglial density was observed after 4 weeks of CUMS exposure compared to the respective control group mice (Table S1L). (**C**) No significant difference in microglial density was observed between the control group mice exposed to acute stress and those not exposed (Table S1M). (**D**) No significant difference was observed after CUMS exposure followed by acute stress re-exposure compared to the respective CUMS group mice without re-exposure (Table S1N). Statistical analysis was performed using two-way ANOVA, followed by Bonferroni’s post-hoc test. Data are presented as the mean ± SEM. Each sample was obtained from at least 18 images (n = 3 for each group); * *p* < 0.05.

Substantial evidence from previous studies has indicated that the medial prefrontal cortex (mPFC) plays a critical role in the regulation of emotional processing and behavioral responses. Dysfunction in the mPFC has been linked to several disorders, including depression and anxiety-related behaviors [31]. To examine changes in microglial activation following different durations of CUMS exposure, we assessed microglial density in the mPFC using immunohistochemical DAB staining for Iba-1 (Figure 6A, top panel; an overview image is shown in Figure S2). Consistent with prior findings, the number of microglia remained stable not only in the hippocampus but also in the mPFC. As expected, no significant differences were observed in the control groups from 2 to 6 weeks (Figure 6B, blue column; detailed in Table S1L). In contrast, a significant increase in microglial density was observed after 4 weeks of CUMS exposure, mirroring changes seen in the hippocampus at the same time point. However, unlike in the hippocampus, no statistically significant differences were observed in microglial density in the mPFC after 2 or 6 weeks of CUMS exposure (Figure 6B, red column; 4 weeks: control = 261.67 ± 12.66 vs. CUMS = 304.67 ± 16.05 cells/mm^2^, *p* = 0.04; mean ± SD). These results suggest that microglial activation in response to chronic stress varies across different depression-related brain regions, such as the hippocampus and mPFC.

Several studies have reported that stress resilience and vulnerability differ across stress-related brain regions, including the hippocampus and mPFC. The hippocampus and mPFC play distinct roles in the stress response-related hypothalamic–pituitary–adrenal (HPA) axis, with the mPFC exerting strong inhibitory control over stress pathways [32]. Furthermore, hippocampal dysfunction has been identified as a major consequence of chronic stress exposure [33]. However, significant differences in microglial density were observed between the hippocampus and mPFC. These findings suggest that while microglial density remains relatively stable within individual brain regions, it varies across different brain areas (“hippocampus”: 322.0 ± 3.0; “mPFC”: 264.0 ± 6.0 cells/mm^2^, t(16) = 8.923, *p* < 0.001; mean ± SD).

### 3.7. The Phenomenon of Microglial Priming in the mPFC Was Not Confirmed After Different Durations of CUMS Exposure

As described in Section 3.5, half of the mice in each group (including the control group) were subjected to 5 h of physical restraint for acute stress re-exposure before tissue collection. Changes in microglial density in the mPFC were evaluated using immunohistochemical DAB staining for Iba-1 (Figure 6A, bottom panel; an overview image is shown in Figure S2). Similarly, no significant changes in microglial density were observed in the mPFC despite exposure to acute stress (physical restraint) in the control group, a pattern similar to what was observed in the hippocampus (Figure 6C; detailed in Table S1M). Furthermore, no statistically significant differences were observed in microglial density in the mPFC between the acute and non-acute stress re-exposure groups after different durations of CUMS exposure (Figure 6D; detailed in Table S1N). These results suggest that the sensitivity of the priming response in microglial activation differs between the hippocampus and mPFC, despite both regions being associated with depression.

### 3.8. The Morphological Changes in Microglia Showed Diverse Fluctuations After Different Durations of CUMS Exposure

**Figure 7:**
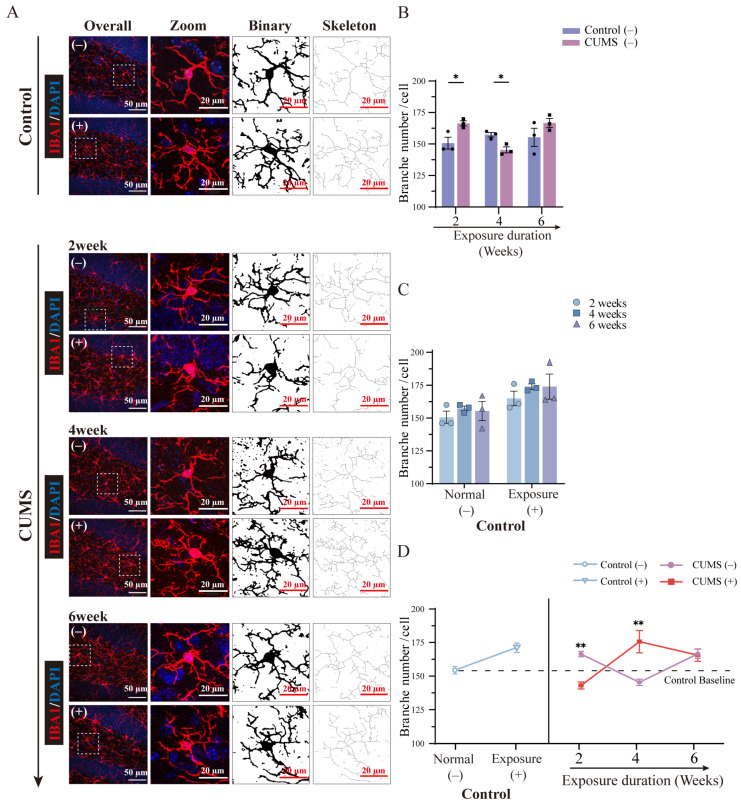
The morphological diversity of microglia was observed after CUMS exposure, followed by acute stress re-exposure. (**A**) IF staining of Iba-1 images showed changes in microglial morphology in the DG of the hippocampus in the control group mice and mice after different durations of CUMS exposure (−). Additionally, changes in microglial morphology after CUMS exposure followed by acute stress (re-)exposure are shown in (+). The images show only the left side of the DG (scale bar = 20 µm). (**B**) The branch number of microglia showed different fluctuation patterns after different durations of CUMS exposure, while no significant difference was observed after 6 weeks of CUMS exposure (Table S1O, n = 3 for each group). (**C**) No significant difference in the branch number of microglia was observed between the control group mice exposed to acute stress and those not exposed (Table S1P, n = 3 for each group). (**D**) After CUMS exposure followed by acute stress re-exposure, the branch number of microglia showed a converse fluctuation pattern compared to those not exposed to acute stress in respective CUMS group mice, while no significant difference was observed after 6 weeks of CUMS exposure followed by acute stress re-exposure (Table S1Q). Statistical analysis was performed using two-way ANOVA followed by Bonferroni’s post-hoc test. Data are presented as the mean ± SEM. Each sample was obtained from at least 18 images; * *p* < 0.05, ** *p* < 0.01.

A substantial number of studies have indicated that microglia continuously monitor the local brain environment, respond sensitively to various stimuli, and synchronously modify their morphological features, such as soma area, branch number, and length [34,35]. This highlights the remarkable morphological diversity of microglia. Moreover, the morphological diversity of microglia in response to stimuli is widely considered to be strongly related to functional changes that help maintain brain homeostasis more efficiently. In our study, we quantitatively analyzed morphological changes in microglia in the hippocampus after different durations of CUMS exposure using immunofluorescence staining for Iba-1 (Figure 7A, no (re-)exposure). Despite the fact that Iba-1 has long been recognized as a valid marker for detecting microglia and assessing microglial morphological changes in both human and rodent experiments, it is important to note that blood-derived myeloid cells (e.g., border-associated macrophages) also express Iba-1. Nevertheless, our results, along with previous studies, support the continued validity of using Iba-1 to detect microglia and assess their morphological changes within the brain parenchyma (e.g., dentate gyrus; see details in Figures S3 and S4).

Several studies have reported a series of morphological changes in microglia after CUMS exposure, such as a reduction in branch number and shortening of branch length [36]. Interestingly, the branch number of microglia significantly increased after 2 weeks of CUMS exposure compared to the respective control group. In contrast, after 4 weeks of CUMS exposure, a noticeable reduction in the branch number of microglia was observed compared to the control group. It has long been considered that, under pathological conditions, ramified microglia, which function as surveillants, exhibit a larger soma area and shorter branching processes, transitioning into amoeboid microglia characterized by strong phagocytosis [37]. However, recent studies have shown that ramified microglia can rapidly increase their branching processes and transition into a hyper-ramified state during or after acute and chronic stress exposure [38]. No statistically significant difference was observed between the 6-week CUMS exposure group and the control group (Figure 7B, blue column; 2 weeks: control = 150.67 ± 6.60 vs. CUMS = 166.33 ± 2.87 number/cell, *p* = 0.01; 4 weeks: control = 157.33 ± 2.49 vs. CUMS = 145.33 ± 3.40 number/cell, *p* = 0.04; 6 weeks: control = 155.33 ± 10.27 vs. CUMS = 166.67 ± 4.92 number/cell, *p* = 0.22; mean ± SD, detailed in Table S1O). These results demonstrate that microglia undergo complex morphological changes in response to different levels of stress during CUMS exposure.

### 3.9. The Microglial Priming-Related Morphological Changes Showed a Converse Pattern Depending on the Duration of CUMS Exposure

As described in Section 3.5, half of the mice in each group (including the control group) were subjected to 5 h of physical restraint for acute stress (re-)exposure before tissue collection, and morphological changes in microglia in the hippocampus were evaluated and compared using immunofluorescence staining of Iba-1 (Figure 7A, (re-)exposure). After 5 h of acute stress exposure, comparisons were made between the control group with acute stress exposure and the group without acute stress exposure. However, regarding the branch number of microglia, no statistically significant difference was observed in any of the control group mice, regardless of whether they were exposed to acute stress (Figure 7C; detailed in Table S1P).

The changes related to the priming reaction of microglial activation were observed not only in the number of microglia but also in the morphological changes in microglia, such as the number of branches. Interestingly, in contrast to the fluctuation pattern in the number of microglia, compared to the 2-week CUMS group without acute stress re-exposure, acute stress re-exposure resulted in a significant decline in microglial branch numbers (Figure 7D, 2 weeks: CUMS (−) = 166.33 ± 2.87 vs. CUMS (+) = 143.0 ± 3.74 number/cell, *p* = 0.010; mean ± SD). In contrast, after 4 weeks of CUMS exposure followed by acute stress re-exposure, the branch number of microglia noticeably increased compared to the non-acute stress re-exposed 4-week CUMS group (Figure 7D, 4 weeks: CUMS (−) = 145.33 ± 3.40 vs. CUMS (+) = 175.67 ± 11.84 number/cell, *p* = 0.001; mean ± SD). Thus, the microglial branches that increased after 2 weeks of chronic stress decreased following acute stress, while those that decreased after 4 weeks of chronic stress increased following acute stress. However, after 6 weeks of CUMS exposure and additional acute stress re-exposure, no significant difference in the branch number of microglia was observed compared to the non-acute stress re-exposed CUMS 6-week group (Figure 7D, 6 weeks: CUMS (−) = 166.67 ± 4.92 vs. CUMS (+) = 165.67 ± 6.60 number/cell, *p* > 0.99; mean ± SD, detailed in Table S1Q). Hence, these results suggest that the changes related to the priming reaction of microglial activation involve not only the number of microglia but also their morphological diversity, such as the branch number of microglia.

## 4. Discussion

In this study, we demonstrate that microglial priming response patterns are influenced by their current activity states, which can either enhance or suppress responses to secondary stimuli, or, if physiological limits are exceeded, prevent priming altogether. By investigating the temporal dynamics of microglial changes in a rodent model of depression using the chronic unpredictable mild stress (CUMS) paradigm, we identified distinct patterns of microglial responses. Short-term CUMS exposure (up to 2 weeks) and subsequent re-exposure to stress induced dynamic morphological changes in microglia, whereas long-term exposure (6 weeks) and re-exposure failed to elicit similar changes, despite the manifestation of depressive-like behavior being evident only after long-term stress. These findings suggest that the fluctuation in microglial priming is regulated by the state of microglial activation, which is dependent on the duration and intensity of chronic stress exposure.

This fluctuation pattern in body weight is consistent with several previous studies that have reported a significant decline in rodent body weight after CUMS exposure [13,39]. However, it is important to note that despite a significant reduction in body weight confirmed after 2 weeks, no significant depression-like behaviors were detected in the TST or FST tests. While body weight reduction may serve as a hallmark symptom of depression-like behavior induced by CUMS exposure, depression-like behavior cannot be solely attributed to body weight reduction alone [40]. Nevertheless, in view of our experimental findings, body weight reduction can be regarded as a systemic physiological response to CUMS exposure, which may in turn contribute to the progression of symptoms. Furthermore, no previous studies have examined fluctuations in body weight following CUMS exposure after behavioral tests assessing depression-like or anxiety behaviors. In most previous studies, mice exposed to CUMS were immediately sacrificed for tissue collection after behavioral tests [36,41]. Therefore, it remains unclear whether body weight was still significantly reduced compared to that in the control group or had recovered to baseline levels. In humans, the pathogenesis of depression is extremely complex and less known; however, it is generally considered to be influenced by multiple factors, such as genetic, social, and environmental factors [42]. Interestingly, proactively changing the current environment appears to facilitate the alleviation of depression-like symptoms in the absence of medicinal treatments by recent research [43].

Our results show that once the CUMS group was no longer exposed to CUMS, a sharp increase in body weight was observed, recovering to levels comparable to the respective control group mice by the end of the behavioral tests, except for the 6-week CUMS group. Furthermore, despite different durations of CUMS exposure, all CUMS groups also showed considerable body weight gain compared to their respective control groups. Hence, these results suggest that the current CUMS paradigm used in this study helps to determine physiological variables in the central nervous system during CUMS exposure, while maintaining relatively stable homeostasis in the whole body and avoiding negative effects from dysfunctions in other organ systems, even after 6 weeks of CUMS exposure. Numerous clinical reports have indicated that persistent weight loss is a characteristic symptom associated with various organ-related disorders [44,45]. Interestingly, regardless of the significant depression-like behaviors already confirmed by TST and FST tests, a significant increase in the body weight of all CUMS groups following the cessation of CUMS exposure was clearly observed. Accordingly, dual evidence has demonstrated a direct causal relationship between body weight reduction and CUMS exposure. However, based on the current results and previous studies, the relationship between significant depression-like behaviors and body weight reduction or increase is merely correlational.

Over the past 20 years, studies have revealed that microglia are not merely “resting cells” involved in immune responses within the central nervous system. Instead, they are highly motile and dynamic, continuously interacting with surrounding neural cells to maintain brain homeostasis under both normal and pathological conditions [46,47]. In recent years, glial–neuronal interactions, including synaptic pruning via glial synapse engulfment (notably by microglia and astrocytes), have increasingly been recognized as essential physiological processes supporting synaptic plasticity [48,49,50]. However, in abnormal situations, such as in various psychiatric disorders like depression, symptom occurrence or progression tends to be accompanied by fluctuations in microglial function [51]. Indeed, the modification of microglial function, including suppression or hyperactivation, can potentially result in neurological dysfunction and contribute to the development of various disorders [52,53]. Additionally, changes in the number or morphology of microglia are widely regarded as being closely related to the state of microglial function, both in normal responses to external stimuli and during the progression of various disorders [35]. However, it is important to note that previous studies have reported several fluctuations in the number and morphological characteristics of microglia during the chronic stress exposure, with some studies even showing opposite trends [36,54,55]. As mentioned above, morphological changes in microglia exhibit extremely dynamic and diverse responses to external stimuli or neuronal activity occurring within timescales of minutes, as observed by in vivo two-photon imaging [56]. Changes in microglial number and morphology also appear to be closely associated with specific stages in the progression of depression, demonstrating the relative stability and distinct characteristics unique to each stage. Furthermore, these changes can indirectly reflect the activity states of microglia across different phases of the progression of depression. Moreover, it is strongly suggested that activated microglia may play different, and sometimes even opposing, roles depending on the duration of chronic stress exposure.

Previous studies have demonstrated that, in the early stages of neural injury, microglia play a neuroprotective role by promptly recognizing and rapidly phagocytosing dying or dead neurons, thereby minimizing the release of neurotoxic substances and limiting further neuronal damage [57]. Consequently, during the early phases of CUMS exposure (2 weeks), microglia exhibit a dual function: suppressing excessive inflammation while supporting injured neurons by releasing neurotrophic factors, such as BDNF. This process comes at the cost of a transient reduction in microglial numbers, reflecting a “self-sacrifice” phenomenon [58]. Consistent with these findings, our results demonstrate a significant reduction in microglial numbers after 2 weeks of CUMS exposure, accompanied by an increase in branch numbers, suggesting that microglia may adapt to the stress environment by enhancing their morphological complexity. However, as CUMS exposure accumulates, microglia undergo sustained activation, eventually disrupting microglial homeostasis and leading to hyperactivation. In line with this progression, we observed a significant increase in microglial numbers and a reduction in branch numbers after 4 weeks of CUMS exposure. This alteration may represent a compensatory mechanism to counteract stress-induced damage (e.g., through phagocytosis), but it may also mark the onset of pathological changes, such as microglial hyperactivation. This state is characterized by an increased number of hyperactivated microglia and excessive secretion of pro-inflammatory cytokines, which may induce neuronal apoptosis and ultimately result in neural dysfunction [59]. In addition to the accumulating evidence from the present and previous studies suggesting changes in microglial activation based on alterations in cell number and morphology during the depression progression, transcriptomic analyses from another study have also revealed characteristic shifts in microglial polarization following CUMS exposure. Specifically, the expression of pro-inflammatory, M1-related gene expression was significantly upregulated, whereas the expression of anti-inflammatory, M2-related gene expression was suppressed [60]. Furthermore, previous studies have indicated that chronic stress-induced microglial hyperactivation may lead to a reduction in microglial numbers and exacerbate the progression of depression [61]. In our study, after 6 weeks of CUMS exposure, microglial numbers were significantly reduced, with no significant changes in branch numbers. This finding further supports the notion that microglial function becomes impaired once the physiological limits are exceeded under prolonged stress exposure.

Alternatively, to further elucidate the dynamic changes in microglial activity during or after repeated chronic stress exposure, a novel concept known as “microglial priming” has been proposed. Recently, several studies have indicated that physiological factors (e.g., aging, inflammatory stimuli, and stress) can maintain microglia in a relatively activated state, increasing their susceptibility to secondary stimuli and subsequently triggering a more rapid and amplified pro-inflammatory response [9,10]. However, our results suggest that microglial activity is unable to be indefinitely enhanced to trigger an exaggerated inflammatory response via microglial priming, as it remains constrained within the physiological limits of microglial function. Notably, after 6 weeks of CUMS exposure, microglial numbers were significantly reduced. This observation aligns with previous findings, further supporting the notion that microglial function becomes impaired when subjected to prolonged stress [61]. Once the physiological limits of microglial function were exceeded, regardless of the duration of CUMS exposure (e.g., 6 weeks), microglial dysfunction appeared to occur, as evidenced by the absence of significant changes in the morphological characteristics of microglia upon acute stress re-exposure. Furthermore, chronic stress has been shown to impair glucocorticoid receptor (GR) signaling, which may play a critical role in initiating microglial priming and altering their functional responses upon subsequent acute stress re-exposure following chronic stress exposure [62].

Previous studies have indicated that microglial dysfunction can be detrimental to brain function and may even lead to behavioral abnormalities [61,63]. Furthermore, when microglial activity has been driven to hyperactivity due to relatively long-term CUMS exposure (e.g., 4 weeks), primed microglia fail to trigger a more intense inflammatory response upon acute stress re-exposure, even showing a decrease in microglial numbers. Hence, this implies that although significant depression-like behaviors were observed in both the 4- and 6-week CUMS groups, the underlying mechanism appears to show essentially different microglial activity states. The former is due to microglial hyperactivation, which may lead to excessive secretion of neuronal toxic factors, resulting in the dysfunction of neuronal activity. In contrast, the latter is attributed to microglial dysfunction, which hinders the maintenance of brain homeostasis and may lead to the absence of debris clearance and neurotrophic factor release, ultimately resulting in neuronal apoptosis [64,65].

Our study sheds light on the dynamic fluctuations in microglial activity states that occur during chronic stress exposure and the potential role of primed microglia in defining the limits on stress resilience and fostering stress vulnerability. These insights may suggest a novel therapeutic approach targeting microglia in depression, where microglial function could be selectively enhanced or inhibited based on activity states at specific stages of depression progression, rather than being indiscriminately suppressed or depleted in the brain [54]. One of the challenges of this study involves not only examining changes in microglial numbers and morphology but also attempting to accurately identify microglial states using molecular markers corresponding to their specific roles. However, future studies should not only focus on morphological alterations but also investigate the molecular and cellular mechanisms underlying microglial dysfunction. In particular, dysregulation of signaling pathways such as the TLR4/NF-κB or MAPK pathways has been implicated in stress-induced neuroinflammatory responses and apoptosis [36,66]. This approach would allow for a more precise understanding of microglial activity under chronic stress. Furthermore, it would be highly intriguing to examine changes in microglial roles and even neuronal activity throughout the duration of chronic stress exposure using the stable and sustainable paradigm of CUMS, beyond its conventional application as a depression model. Nevertheless, one limitation of this study lies in the relatively small sample size used for behavioral and immunohistochemical analyses, which was primarily due to logistical and ethical constraints. While this may have limited the statistical power and thereby contributed to the absence of statistical significance in some results (e.g., Section 3.5 on microglial number changes in the mPFC after 6 weeks of CUMS exposure), the observed trends remain consistent across all results in our study, supporting the overall reliability of our findings. Future studies with increased sample sizes are planned to validate and extend the current findings, which will help strengthen the reliability of the conclusions and allow for more definitive statistical analysis.

## 5. Conclusions

In conclusion, our data suggest that microglial priming responses are state-dependent, either enhancing or suppressing secondary stimulus responses. However, once they exceed physiological limits, further activation is prevented. This study highlights the necessity of a more comprehensive understanding of the varied effects on brain homeostasis caused by fluctuations in microglial function across different stages of depression progression induced by chronic stress exposure. Moreover, future research should further explore the relationship between changes in the physiological function of primed microglia and increased stress vulnerability under varying durations of chronic stress exposure.

## Figures and Tables

**Table 1:** Chronic unpredictable mild stressors.

Stressor	Description
**Tail pinch**	A steel binder clip (20 mm opening width, KOKUYO, Osaka, Japan) was placed 4 cm from the tip of the tail for 2 min (after each clip was removed, the steel binder clip was disinfected with 70% alcohol).
**4 °C water stress**	About 1 L of 4 °C tap water was poured into the cage (27.0 cm length, 16.0 cm width, 11.5 cm height) and maintained at a water depth of approximately 3 cm. The CUMS group mice were placed in this cage for 2 min.
**Cage oscillation**	The CUMS group mice were placed in the cage, which was fixed on top of the shaker for 30 min (each oscillation speed was kept the same, Double-Action Lab Shaker SRR-2, AS ONE Corp., Osaka, Japan).
**Offensive odor**	2,4,5-Trimethylthiazole (TMT) is a secretion oozed by a natural rodent predator. Four µL TMT was dropped onto the filter paper, which was placed in the center of the cage, and the CUMS group mice were placed in this cage for 30 min.
**Physical restraint**	Each mouse in the CUMS group was stuffed into the polyethylene disposable mouse restrainer for 3 h (15.5 cm length, the width of topside opening is 1 cm, the radius of Bottom opening is 13 cm, MDC-20, Nihon Bioresearch Inc., Gifu, Japan).
**Overnight illumination**	The light/dark cycle was disrupted (e.g., during the dark-cycle period, illumination for 12 h).
**Water deprivation**	The bottle of drinking water was removed for 15 h. However, food was provided as usual.
**Food deprivation**	The food was removed for 15 h. Drinking water was provided as usual.
**Flash stress**	The CUMS group mice were subjected to flash stress for 24 h (the two flash LED devices were placed on the left and right of the home cage). The strobe speed was set as the fastest (S81 LED II, ADJ Products, LLC, Los Angeles, CA, USA). Food and water were provided during this period.
**Damp sawdust**	A total of 400 mL tap water was poured into 20 g sawdust in the cage, and the CUMS group mice were placed in the cage for 24 h (tap water was at room temperature, food and water were provided during this period).
**Cage tilting**	The cage was tilted forward by 45 degrees for 24 h (food and water were provided during this period).

**Table 2:** CUMS Stress Performed Frequency Per Week.

Stressor	Period of Exposure	Times of Exposure
**Tail pinch**	2 min	4
**4 °C water stress**	2 min	2
**Cage oscillation**	30 min	3
**Offensive odor**	30 min	3
**Physical restraint**	3 h	3
**Overnight illumination**	12 h	2
**Water deprivation**	15 h	1
**Food deprivation**	15 h	1
**Flash stress**	24 h	1
**Damp sawdust**	24 h	1
**Cage tilting**	24 h	1

## Data Availability

All study data are included in the main text and the Supporting Information. Any additional information required to reanalyze the data reported in this paper is available from the lead contact upon request. Further information and requests for resources, reagents, and codes (Python and ImageJ macro) should be directed to and will be fulfilled by the lead contact, Dr. Nobuhiko Kojima (kojima033@toyo.jp).

## Supplementary Materials

The following supporting information can be downloaded at: https://www.mdpi.com/article/10.3390/brainsci15050534/s1. Refs. [67,68,69,70,71] are cited in the supplementary Materials.
