# Supplementary material for: Chronic Stress Modulates Microglial Activation Dynamics, Shaping Priming Responses to Subsequent Stress
Source: Brain Sci. 2025 May 21;15(5):534. doi: 10.3390/brainsci15050534 (PMC12110633; doi:10.3390/brainsci15050534)
Supplement: Supplementary file 1 [file brainsci-15-00534-s001.zip › brainsci-3630491-Supplementary.pdf]

brain sciences

MDPI

Chronic Stress Modulates Microglial Activation Dynamics, Shaping Priming Responses to Subsequent Stress

Junyu Chen[a,b,†], Jiacheng Huang[a,c,†], Taolei Han[a], Nobuhiko Kojima[a,d*]

[a] *Laboratory of Molecular neurobiology, Faculty of Life sciences, Toyo university, Saitama 352-8510, Japan*

[b] *Super-network Brain Physiology, Graduate School of Life Sciences, Tohoku University, Sendai 980-8577, Japan*

[c] *Department of Histology and Cell Biology, Graduate School of Medical Sciences, Kanazawa University, Ishikawa 920-8640, Japan*

[d] *Research Center for Biomedical Engineering, Toyo University, Saitama 352-8510, Japan*

[*] Corresponding author.

E-mail address: kojima033@toyo.jp (N. Kojima).

[†]These authors contributed equally to this work and share first authorship.

This document includes Supplementary Methods, Supplementary Figures S1–S4 and Supplementary references.

1 | Supplemental Methods

S1.1 | Immunohistochemical assay

The animals were anesthetized with an injection of sodium pentobarbital (150 mg/kg, i.p.) and sacrificed after all tests. Intracardial perfusion with phosphate-buffered saline (PBS) containing 0.5% heparin was performed to remove blood, and the brains of the mice were fixed with 4% paraformaldehyde (PFA). The fixed brains were sectioned into coronal slices (40 µm thick) using a vibratome (LEICA VT 1200S). Slices containing the medial prefrontal cortex (mPFC) and hippocampal regions were collected and stored in 24-well plates with 0.05% NaN_3 in 1 × PBS at 4 °C.

S1.1.1 | Immunofluorescence staining with Iba1, TMEM119, CD206 antibodies.

Iba1 is a commonly marker for microglia and macrophages [**67**]. Recent reports have shown that in addition to the parenchymal microglia in the central nervous system (CNS), there are also non-parenchymal macrophages (border-associated macrophages, BAM) located at CNS borders (including the choroid plexus, perivascular space, and meninges), which specifically express CD206 [**69**]. To better distinguish microglia from BAM, TMEM119, a marker that is specifically and stably expressed in microglia of both mice and humans, has been used in combination with CD206 for colocalization analysis [**70,71**]. Accordingly, sections containing the hippocampus (dentate gyrus, DG) and perivascular area were blocked with M.O.M Mouse IgG Blocking Reagent (Vector Labs, Cat.No.BMK-2202) containing 0.1% Triton X-100 and 0.05% NaN3 in 1 × PBS for 1 h at RT. They were then incubated overnight at 4 ºC with the primary antibody, rabbit anti-Iba1 (Wako Chemicals, Cat.No.019-19741, 1:800), mouse anti-TMEM119 (Synaptic Systems, Cat.No.400011, 1:400), rat anti-CD206 (BioLegend, Cat.No.141701, 1:200). The secondary antibody, donkey anti-rabbit IgG (H+L) Alexa Fluor® 594 (Invitrogen, Cat.No.A21207, 1:200), goat anti-rat IgG (H+L) Alexa Fluor® 488 (abcam, Cat.No.ab150157, 1:200), donkey anti-mouse IgG (H+L) Alexa Fluor® 405 (abcam, Cat.No.ab175658, 1:200) was applied for 1 h at RT. Images were obtained using a laser scanning confocal super-resolution microscope (SpinSR10; OLYMPUS, Japan), and analyzed with the Plot Z-Axis Profile plugin in Fiji/ImageJ. Fluorescence intensity profiles were generated and further analyzed using AxoGraph (AxoGraph Scientific, Australia).

S1.1.2 | Immunofluorescence staining with Iba1, TMEM119 antibodies.

Based on antibodies characterization described above, to validate that Iba1-positive cells in the dentate gyrus (DG) and prefrontal cortex (mPFC) regions are highly specific to microglia. Sections containing the DG and mPFC were blocked with M.O.M Mouse IgG Blocking Reagent (Vector Labs, Cat.No.BMK-2202) containing 0.1% Triton X-100 and 0.05% NaN3 in 1 × PBS for 1 h at RT. They were then incubated overnight at 4 ºC with the primary antibody, rabbit anti-Iba1 (Wako Chemicals, Cat.No.019-19741, 1:800), mouse anti-TMEM119 (Synaptic Systems, Cat.No.400011, 1:400). The secondary antibody, donkey anti-rabbit IgG (H+L) Alexa Fluor® 594 (Invitrogen, Cat.No.A21207, 1:200), donkey anti-mouse IgG (H+L) Alexa Fluor® 405 (abcam, Cat.No.ab175658, 1:200) was applied for 1 h at RT. Subsequently, 4’6-diamidino-2-phenylindole (DAPI, 1:1000) was added and incubated for 15 min at RT. Images were obtained using a laser scanning confocal super-resolution microscope (SpinSR10; OLYMPUS, Japan), and analyzed with the Plot Z-Axis Profile plugin in Fiji/ImageJ. Fluorescence intensity profiles were generated and further analyzed using AxoGraph (AxoGraph Scientific, Australia). Correlation analysis of the obtained data was performed using GraphPad Prism software (version 10.0, USA) via simple linear regression, with the R^2 value.

2 | Supplemental Figures

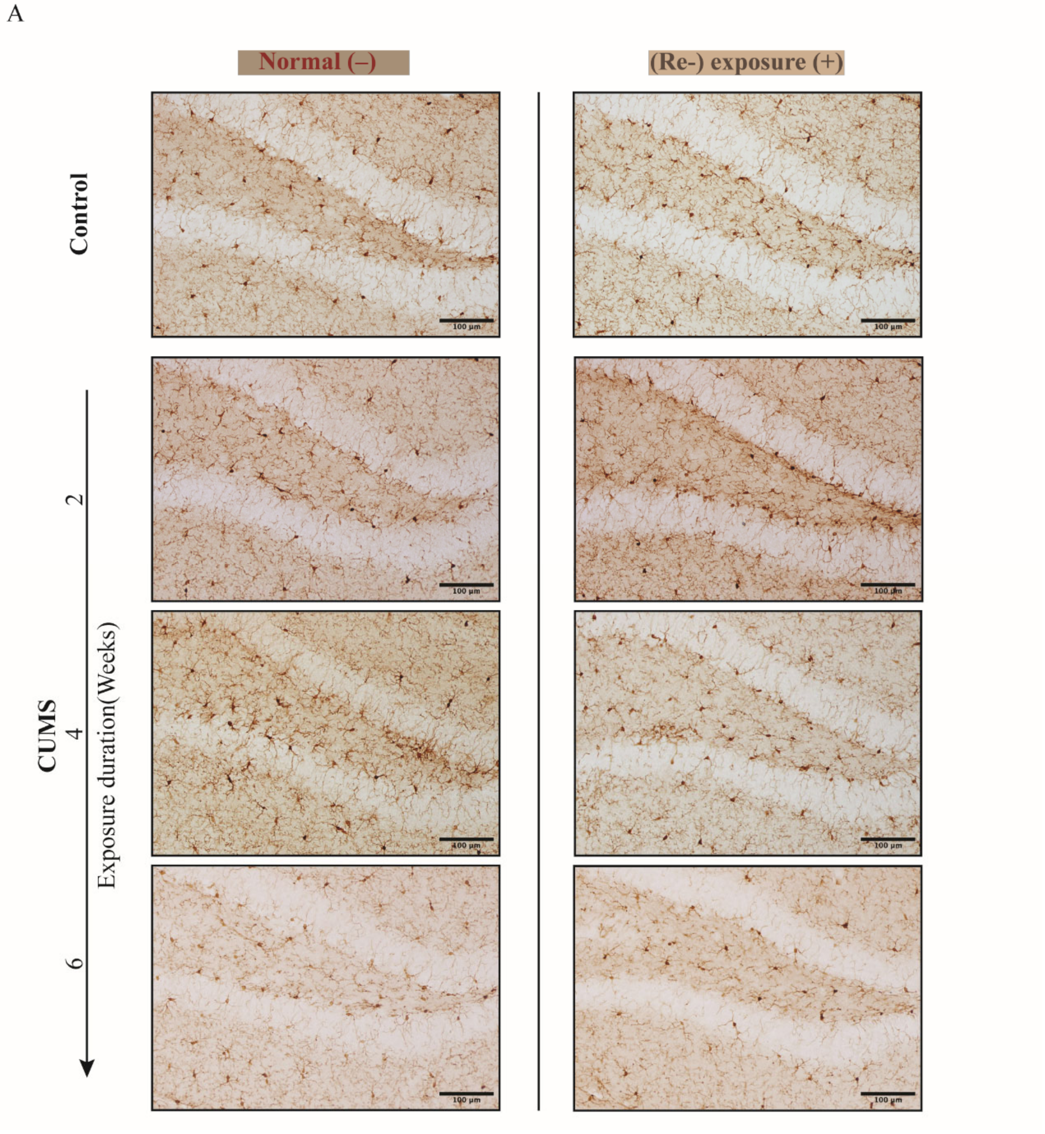

Figure. S1. Dynamic variations in hippocampal microglial density following different durations of CUMS exposure and acute stress re-exposure (A) DAB staining of Iba-1 images showed changes in microglial density in the DG of the hippocampus of the control group mice and mice after different durations of CUMS exposure (left panel). In addition, changes in microglial density after CUMS exposure followed by acute stress (re) exposure are shown in (right panel). Images show only the left side of the DG, n = 3 for each group; scale bar = 100 μm.

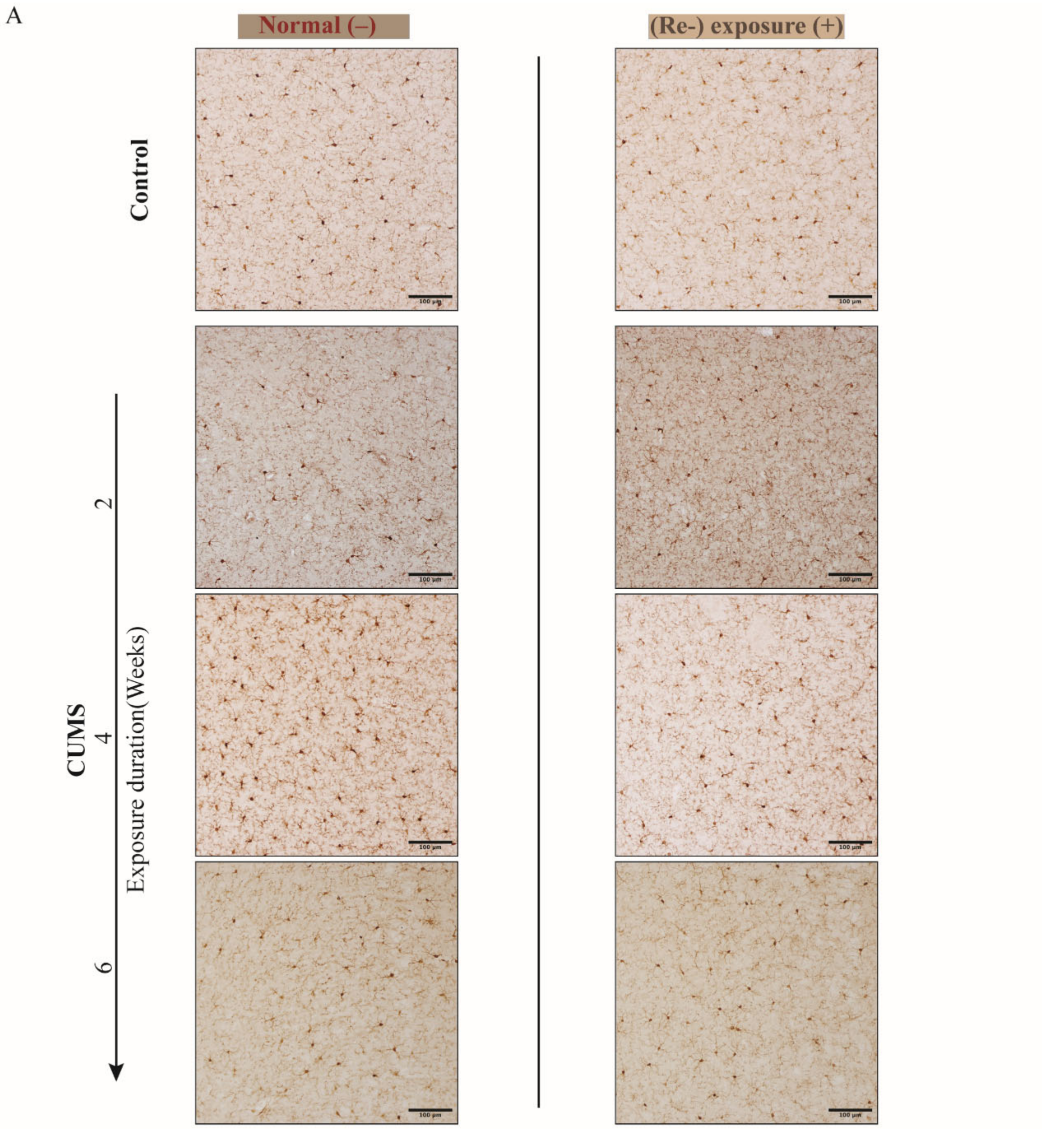

Figure. S2. Dynamic variations of microglial density in mPFC following different durations of CUMS exposure and acute stress re-exposure (A) DAB staining of Iba-1 images showed changes in microglial density in the mPFC of the control group mice and mice after different durations of CUMS exposure (left panel). In addition, changes in microglial density after CUMS exposure followed by acute stress (re) exposure are shown in (right panel). Scale bar = 100 µm.

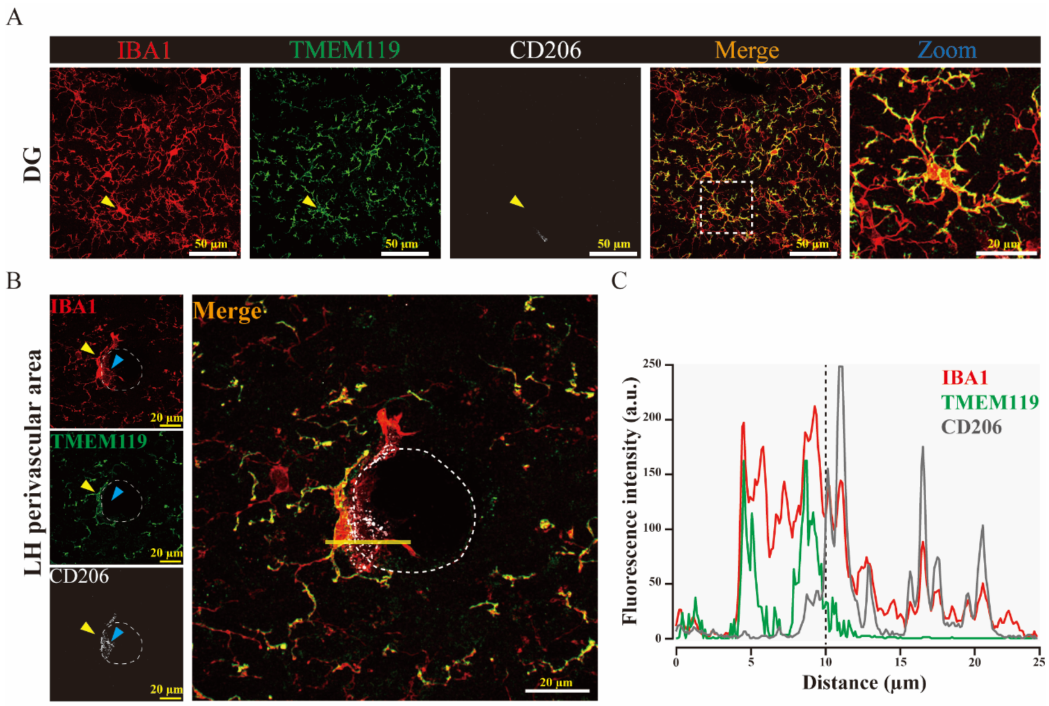

Figure. S3. Transmembrane protein 119 (TMEM119) has been identified as a specific marker for microglia, with its expression not observed in other blood-derived myeloid cells infiltrating the CNS. (A) Immunofluorescent staining for Iba1 (red) and TMEM119 (green) revealed that the majority of IBA1$^+$ cells (especially be regard as parenchymal microglia) co-expressed the microglial marker TMEM119 in the dentate gyrus (DG). In contrast, the fluorescence signal for CD206 (white), a mannose receptor typically expressed on anti-inflammatory (M2) macrophages, was nearly undetectable in the DG (yellow arrows). **(B)** Triple immunofluorescent staining for IBA1 (red), TMEM119 (green), and CD206 (white) revealed that IBA1$^+$/TMEM119$^+$ cells, which are primarily considered vessel-associated microglia (VAMs), did not express CD206 (yellow arrows). In contrast, IBA1$^+$/CD206$^+$ cells, which are primarily identified as perivascular macrophages (PVMs), lacked TMEM119 expression (blue arrows). The region outlined by the white dotted line indicates a blood vessel. A fluorescence intensity line profile along the yellow line in the merged image (left) is shown on the right. **(C)** Colocalization analysis of IBA1 (red), TMEM119 (green), and CD206 (dark gray) showed that IBA1$^+$ cells overlapped with both TMEM119$^+$ and CD206$^+$ signals, indicating that IBA1$^+$ cells can represent either microglia or perivascular macrophages, particularly in perivascular area. In contrast, TMEM119$^+$ signals were only colocalized with IBA1$^+$ but not with CD206$^+$, suggesting that TMEM119 is able to be recognized as a specific marker for microglia. The scale bar in the overall image represents 50 μm, and in the zoomed-in image represents 20 μm.

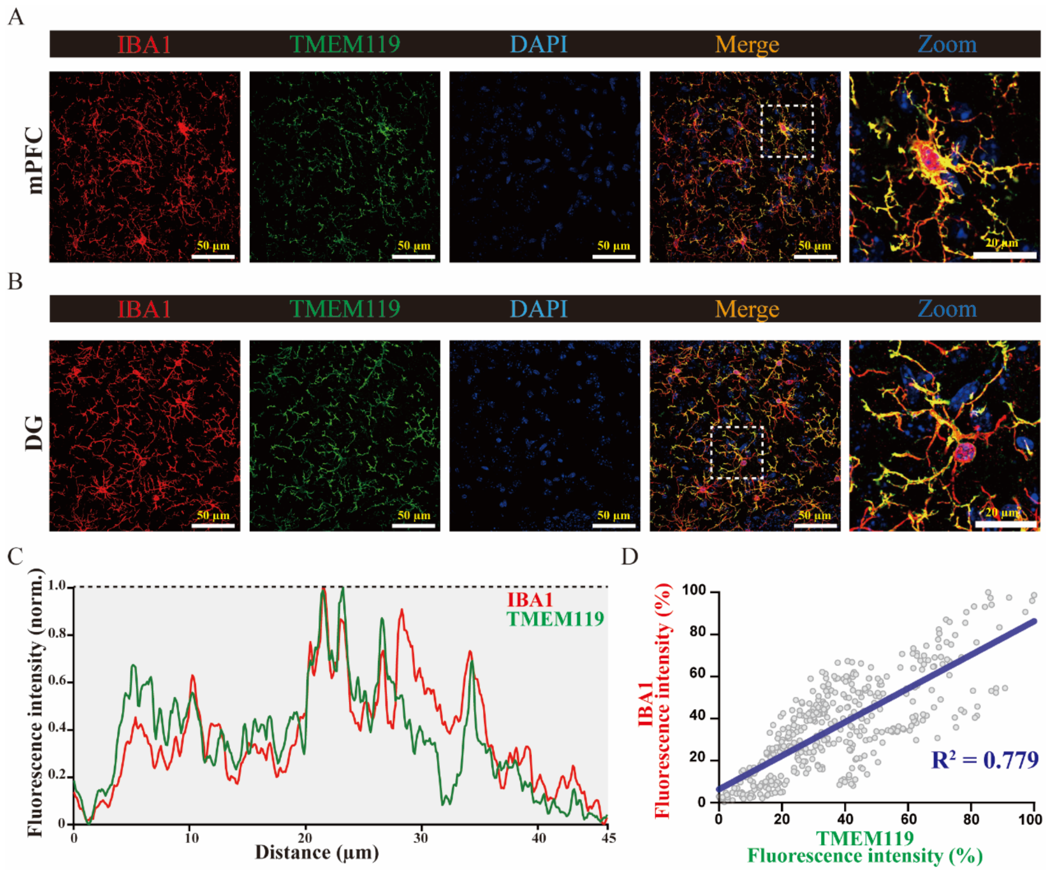

Figure. S4. The signal of IBA1 highly colocalized with the signal of TMEM119 in brain parenchyma, indicating that IBA1 marker still able to be recognized as appropriate marker for parenchymal microglia. (A–B) Immunofluorescent staining for IBA1 (red) and TMEM119 (green) revealed a high degree of colocalization between Iba1 and TMEM119 signals in both the dentate gyrus (DG) and the medial prefrontal cortex (mPFC). **(C–D)** Line profile analysis in the zoomed-in image of the DG revealed that IBA1 (red) and TMEM119 (green) signals were closely colocalized throughout the analyzed region. Consistently, this colocalization was further confirmed by fluorescence intensity analysis of the two markers ($R^2 = 0.7788$, XY log = 425, $p = <0.001$). The scale bar in the overall image represents 50 µm, and in the zoomed-in image represents 20 µm.

3 | Supplemental References

(67) Sasaki, Y.; Ohsawa, K.; Kanazawa, H.; Kohsaka, S.; Imai, Y. Iba1 Is an Actin-Cross-Linking Protein in Macrophages/Microglia. *Biochemical and Biophysical Research Communications* **2001**, *286* (2), 292–297. https://doi.org/10.1006/bbrc.2001.5388.

(68) Kierdorf, K.; Masuda, T.; Jordão, M. J. C.; Prinz, M. Macrophages at CNS Interfaces: Ontogeny and Function in Health and Disease. *Nat Rev Neurosci* **2019**, *20* (9), 547–562. https://doi.org/10.1038/s41583-019-0201-x.

(69) Utz, S. G.; See, P.; Mildenberger, W.; Thion, M. S.; Silvin, A.; Lutz, M.; Ingelfinger, F.; Rayan, N. A.; Lelios, I.; Buttgereit, A.; Asano, K.; Prabhakar, S.; Garel, S.; Becher, B.; Ginhoux, F.; Greter, M. Early Fate Defines Microglia and Non-Parenchymal Brain Macrophage Development. *Cell* **2020**, *181* (3), 557-573.e18. https://doi.org/10.1016/j.cell.2020.03.021.

(70) Bennett, M. L.; Bennett, F. C.; Liddelow, S. A.; Ajami, B.; Zamanian, J. L.; Fernhoff, N. B.; Mulinyawe, S. B.; Bohlen, C. J.; Adil, A.; Tucker, A.; Weissman, I. L.; Chang, E. F.; Li, G.; Grant, G. A.; Hayden Gephart, M. G.; Barres, B. A. New Tools for Studying Microglia in the Mouse and Human CNS. *Proc. Natl. Acad. Sci. U.S.A.* **2016**, *113* (12). https://doi.org/10.1073/pnas.1525528113.

(71) Satoh, J.; Kino, Y.; Asahina, N.; Takitani, M.; Miyoshi, J.; Ishida, T.; Saito, Y. TMEM119 Marks a Subset of Microglia in the Human Brain. *Neuropathology* **2016**, *36* (1), 39–49. https://doi.org/10.1111/neup.12235.
